# Changes in Health-Related Behaviours Among Adults Who Accessed Real-World Weight Management Support: 12-Month Outcomes

**DOI:** 10.7759/cureus.95035

**Published:** 2025-10-21

**Authors:** Jennifer Kent, Josef Toon, Sarah-Elizabeth Bennett, Laura Holloway, Carolyn Pallister, Jacquie Lavin, Jemma Donovan, Amanda Avery

**Affiliations:** 1 Nutrition, Research and Health Policy, Slimming World, Derby, GBR; 2 Division of Psychology, De Montfort University, Leicester, GBR; 3 Division of Food, Nutrition and Dietetics, University of Nottingham, Nottingham, GBR

**Keywords:** behaviour change, diet quality, healthy lifestyle intervention, obesity, weight management

## Abstract

Background

Large weight losses are desirable, but their benefits are short-lived without sustained behaviour changes that can be maintained at the household level. This longitudinal study, conducted in a real-life setting, investigated changes in weight, dietary habits, activity levels, and physical and mental well-being of members of a community weight management programme (Slimming World), compared with a matched cross-sectional reference group from the general population. The wider influence on the dietary and activity habits of family members was also explored.

Methods

Longitudinal data were collected from members at 0-4 weeks (T1), 3 months (T2), and 12 months (T4) after joining. The reference group completed surveys at each time point. Diet quality scores (NDQS) were calculated using a validated tool, hours of moderate-intensity physical activity were recorded, and mental well-being was assessed using adapted items from the SF Health Survey. Changes in members’ behaviours and comparisons with the reference group were analysed using within- and between-group ANOVAs with p-adjusted post-hoc comparisons.

Results

Of the 1,884 members who provided baseline data, 174 (7.5% male) completed surveys at T1, T2, and T4. At baseline, mean BMI and age were 34.7 ± 7.0 kg/m² and 53.0 ± 12.0 years, respectively. Mean weight change at 12 months was -7.5%. Member NDQS increased from baseline to T1 (11.5 ± 3.2 vs 14.1 ± 2.4, p < 0.001), T2 (14.3 ± 2.7), and T4 (14.1 ± 2.9) (both p < 0.05). Physical activity increased between T1 and T2 (4.6 ± 7.4 vs 6.7 ± 10.1 hours/week, p < 0.01) and was maintained at T4 (6.5 ± 6.0, p < 0.05). At T1, T2, and T4, members had higher NDQS, greater levels of physical activity (p < 0.001), and higher mental well-being scores (p < 0.05) than the reference group. At T2, of the 122 members living with a partner, 79.5% reported influencing their partner to eat healthier meals. Among the 47 members living with children, 66.0% reported influencing them to eat healthier meals. The positive influence on partners’ and children’s eating behaviours observed at T2 remained stable at T4 (both p > 0.05). At T2, 40.7% of members reported encouraging others in their household to become more active, and this proportion remained consistent at T4 (40.5%, p > 0.05).

Conclusion

Although the low response rate across all three surveys is a limitation, the findings suggest that Slimming World’s behaviour change programme is effective in supporting adults (mainly females) living with obesity to make health-related behaviour changes. Members achieved clinically significant weight loss and improvements in diet quality, physical activity, and mental well-being compared with the reference group. These changes were maintained at 12 months, with an additional positive influence reported on family members’ lifestyle habits.

## Introduction

The evaluation of any weight management intervention needs to consider more than just the weight changes that may be achieved. Large weight losses may be desirable and confer short-term health benefits, but it is important that negative long-term health consequences do not occur as a result of any associated nutritional deficiencies arising from the intervention.

The modification of ‘low-risk lifestyle factors’, such as diet, alcohol intake, sedentary behaviours, and physical activity levels, can have long-term physical and mental health benefits [1-3]. These benefits may also extend to others if the healthier changes are adopted by the whole family.

The reality is that adherence to healthy eating guidelines and recommendations for physical activity levels in the UK remains extremely poor [4, 5]. The benefits of having a healthier diet and being less sedentary are being undermined by some of the newer obesity treatment options, albeit evidence-based nutritional and lifestyle strategies have recently been recognised as important in addressing key challenges surrounding GLP-1 treatment of obesity [6].

Interventions that guide individuals towards healthier lifestyle choices and, importantly, empower them to maintain positive behaviour changes in the long term are vital, alongside healthy weight management, to improve health, reduce morbidity, and increase healthy life expectancy. Furthermore, the management of obesity requires behavioural support that is accessible to people within the community and can be integrated into their daily routines.

There is currently very limited ‘real-life’ evidence examining how weight management interventions can support both weight loss and improvements in physical and mental well-being through lifestyle modifications in line with national guidelines. The most recent review of randomised controlled trials of behavioural weight management interventions for adults with a BMI ≥25, delivered in primary care and compared with no or minimal treatment, reported only anthropometric changes (weight and waist circumference) at 12- and 24-month follow-up [7].

This longitudinal study aimed to report weight changes and investigate behavioural changes in dietary habits and diet quality, sedentary behaviours and activity levels, physical health, and mental well-being of Slimming World members and their families, compared with cross-sectional reference samples from the general population, at regular time points over a 12-month period. The null hypothesis was that there would be no difference in weight change, dietary habits, physical activity levels, sedentary behaviours, and mental well-being between the two groups at 12 months.

## Materials and methods

Recruitment

Slimming World is the largest UK-based commercial weight management organisation, established over 55 years ago, that provides adult weight management support through weekly community-based groups held widely across the United Kingdom and the Republic of Ireland. They also offer a digital support programme that can be accessed globally. The programme has previously been shown to help self-funded service members achieve modest but clinically significant weight loss [8] and deliver long-term effective outcomes [9]. The compassion-based behaviour change programme uses a multi-component approach and encourages members, with a range of different health conditions, to adopt a healthy, balanced diet and gradually increase physical activity levels [10]. Members are encouraged to set their own personal target weight and can choose to access support for as long as they wish. Those with health conditions are advised to seek additional support from their healthcare professional team, with referral schemes available in some areas. Given the number of adults supported each week, it is appropriate to evaluate whether the support offered has an impact on dietary habits and physical activity levels, alongside changes in weight and mental well-being.

To be eligible for the study, Slimming World members had to be 18 years or older, not pregnant, and have joined between September 9, 2019 and October 10, 2019, with no history of membership within the previous 12 months. Eligible participants were contacted via email about the aims of the study and were offered the opportunity to be entered into a prize draw to win a high street shopping voucher (funded by Slimming World) after completing each survey. Interested members were provided with a digital participant information sheet and gave consent to participate via the consent form at the start of the survey.

At the baseline survey, participants were asked to retrospectively self-report behaviours prior to joining (T0) and report data from within the first four weeks of membership (T1). Follow-up surveys were sent to all participants who completed the baseline survey to capture longitudinal data at 3 months (T2), 6 months (T3), and 12 months (T4), regardless of whether they remained active members at these subsequent time points. Qualtrics (Qualtrics, Provo, UT, USA) was used to recruit participants from the general population who identified as individuals wanting to lose weight but were not actively managing their weight with a commercial organisation at each time point. A protocol designed to match participants to the demographics of the Slimming World membership included gender (7% male, 93% female) and target sample sizes for each age group category (18-24 years: 10%; 25-34 years: 25%; 35-44 years: 20%; 45-54 years: 25%; 55-80 years: 20% ± 2% for each category).

The 6-month (T3) survey coincided with COVID-19 lockdown restrictions in the UK, and these data have been reported previously. They were excluded from this analysis given the immediate impact of COVID-19 on people’s ability to make lifestyle changes [11].

Questionnaire

Surveys were distributed via Qualtrics (Qualtrics, Provo, UT, USA) and, at each time point, collected data on demographics; self-reported height and weight; general health; dietary intake and behaviours; alcohol consumption; physical activity levels; mental well-being; and wider family influence.

Detailed questions were included to obtain information on health-related dietary behaviours, such as fruit and vegetable consumption (“How many portions did you eat yesterday?”, with examples of portion sizes equivalent to 80 g provided for reference); frequency of sugary drink and wholegrain consumption (“In a typical week, how often do you do the following?”, asked on a 5-point scale ranging from “more than once a day” (5 points) to “never/occasionally” (1 point]); frequency of fatty and sugary food intake (“In a typical month, how often do you do the following?”, asked on a 5-point scale ranging from “once a day or more” to “once a month or less,” with examples of foods high in fat and sugar provided for reference); and alcohol intake (“How many alcoholic drinks do you generally consume each week?”, split by type and volume of drink to enable units of alcohol to be derived).

Diet quality was assessed using the short version of the validated Nutrient-based Diet Quality Score (NDQS) tool, based on fruit, vegetable, wholegrain, and sugary drink consumption, with a higher score indicating greater diet quality [12].

Questions on physical activity and sedentary behaviour were included to identify health-related lifestyle habits. Data on the type, duration, and intensity of activity were obtained, and total hours of moderate-intensity physical activity were calculated using the Sport England Active Lives methodology [13].

The wider influence of participants’ Slimming World membership was also assessed. Members who did not live alone were asked whether they had influenced members of their household to eat healthier meals and/or become more physically active as a result of their membership, at surveys T2 and T4.

A mental well-being score was calculated by adapting items from the Short Form Health Survey [14] to assess how often participants felt calm and peaceful (item 1), had a lot of energy (item 2), felt downhearted and low (item 3), and had been a happy person (item 4). Three additional questions assessed how often participants had been in a sociable mood (item 5), felt stressed (item 6), and felt anxious (item 7). All items were scored from 6 (“All of the time”) to 1 (“None of the time”). A total well-being score (minimum = 7, maximum = 42) was calculated by summing all items after reversing those measuring negative affect (items 3, 6, and 7), with a higher score indicating more positive well-being.

The scale assessing well-being was tested for reliability in multiple ways. The average item-total correlation was high (r = 0.77), and split-half reliability indicated high internal consistency (r = 0.84). Cronbach’s alpha analysis also demonstrated high internal consistency (α = 0.88), with no items identified as improving the scale if removed and a high inter-item correlation (r = 0.52).

Participants

**Figure 1:**
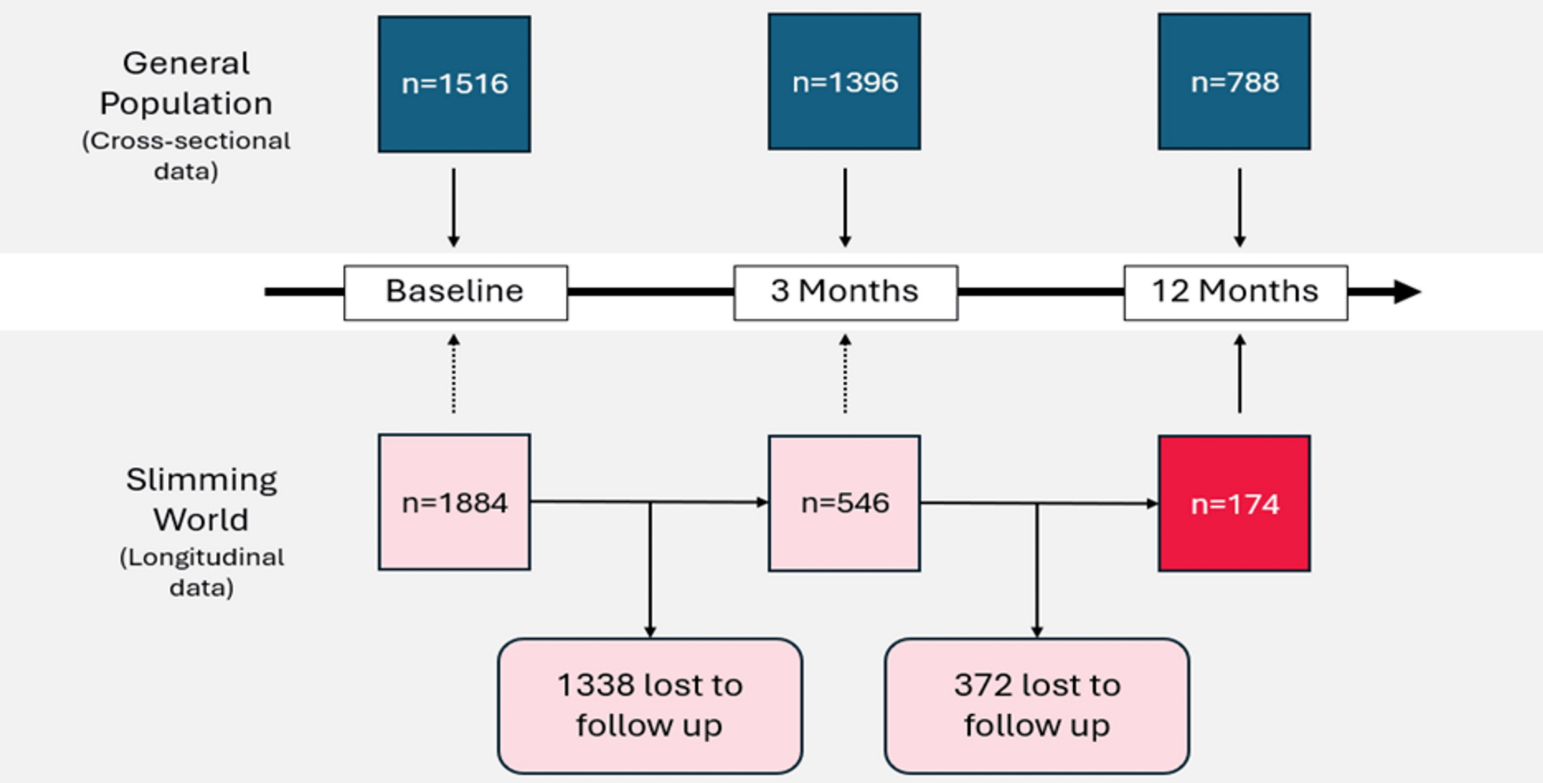
Participant recruitment.

This paper presents longitudinal data from the 174 Slimming World members who provided responses at baseline (T1), 3 months (T2), and 12 months (T4) (Figure 1). The cross-sectional data from samples of the general population served as a reference group of adults living with overweight or obesity who were not actively managing their weight with a commercial weight management provider at the time of survey completion.

Statistical analysis

All statistical analyses were conducted using R (version 4.1.0; R Core Team, 2021). No adjustments were made for covariates (age or gender); only comparisons of scores for each variable were performed. Within-group differences in the longitudinal data from members were analysed using repeated-measures ANOVA, followed by Bonferroni-adjusted post-hoc comparison tests to assess the main effects of time point.

As cross-sectional data were collected from different samples of the general population, between-group differences were analysed using one-way independent ANOVA to compare means at each time point, with adjusted post-hoc tests to examine any main effects between groups. Where data were collected as proportions, comparisons between members and the general population were conducted using z-proportion tests.

Data in the text are presented as mean ± SD, and error bars within figures represent standard deviation. Significance levels are indicated as *p < 0.05, **p < 0.01, ***p < 0.001.

Ethics

The study was performed in accordance with the Declaration of Helsinki and approved by the University of Nottingham, School of Biosciences Ethics Committee (No: SB1819/36).

## Results

Baseline characteristics

Baseline characteristics of participants who completed the surveys at T1, T2, and T4 are reported in Table 1. The 174 members who completed all surveys (T1, T2, and T4) were older (H(3) = 116, p < 0.001) and had a higher BMI than each of the general population groups (H(3) = 94.4, p < 0.001), and were also older and heavier compared to the 1,884 members who only completed the survey at T1. Of the Slimming World members, 161 were female. Proportion by gender varied significantly across the four samples (χ²(3, 3857) = 14.2, p < 0.01). Comparison of standardised residuals revealed that this significance was driven by the greater proportion of males within the general population group at T1.

**Table 1:** Characteristics of Slimming World (SW) members compared with the general population at each time point.

	SW T1	SW T1, T2 & T4	General population		
			T1	T2	T4
Number of participants	1,884	174	1,516	1,396	788
Gender (% female / % male)	93.9 / 6.1	92.5 / 7.5	89.6 / 10.4	92.6 / 7.4	93.5 / 6.5
Mean ± SD age (years)	47.7 ± 13.3	53.0 ± 12.0	44.5 ± 14.5	47.6 ± 12.0	49.2 ± 13.5
Mean ± SD weight (kg) (self-reported)	92.7 ± 19.7	94.0 ± 18.9	82.3 ± 19.7	81.2 ± 19.7	80.7 ± 17.6
Mean ± SD BMI (kg/m²)	33.8 ± 6.65	34.7 ± 7.0	30.2 ± 6.8	30.3 ± 7.1	30.7 ± 6.3

Of the 174 Slimming World participants, 90.2% (n = 157) and 78.7% (n = 137) were still active members at T2 and T4, respectively.

Self-reported weight data

**Figure 2:**
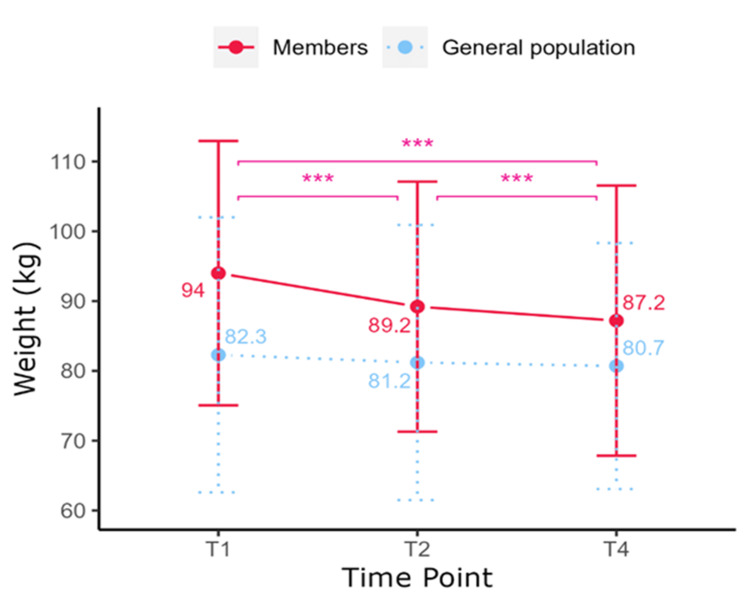
Self-reported weight (kg) of Slimming World (SW) members and the general population across time points. *p < 0.05, **p < 0.01, ***p < 0.001.

Between T1 and T4, members achieved a mean weight loss of 6.8 ± 9.9 kg, which equated to a weight change of -7.5 ± 8.9% and a mean change in BMI of -2.6 ± 3.2 kg/m². Weight changed over time (F(1.3, 218.22) = 75.3, p < 0.001), as shown in Figure 2. Post-hoc tests revealed decreases between T1 and T2 (94.0 ± 18.9 kg vs 89.2 ± 17.9 kg, p < 0.0001) and between T2 and T4 (89.2 ± 17.9 kg vs 87.2 ± 19.3 kg, p < 0.0001). Weight did not differ between the general population at T1, T2, and T4 (F(2, 3696) = 1.9, p = 0.14).

General health

At T1, 20.8% of members reported that their general health had improved compared to one year previously. This increased at T2 (49.1% vs 20.8%; T2 vs T1, p < 0.001) and was sustained at T4 (49.7%; T4, p > 0.05). The proportion of members who reported improved general health compared to one year previously did not differ from the general population at T1 (20.8% vs 18.3%, p > 0.05). However, more members reported improved general health compared to the general population at both T2 (49.1% vs 18.6%, p < 0.001) and T4 (49.7% vs 17.8%, p < 0.001).

Dietary changes

Daily Fruit and Vegetable Intake

Over time, there was a significant change in members’ daily fruit and vegetable intake (F(2.8, 480.8) = 50.6, p < 0.0001). Post-hoc comparisons showed that the number of fruit and vegetable portions consumed by members increased soon after joining (7.3 ± 2.8 vs 4.8 ± 3.0 portions; T1 vs T0, p < 0.001) and remained higher than before joining at T2 (7.4 ± 2.8 portions, p < 0.0001) and T4 (7.0 ± 3.1 portions, p < 0.001).

There were no significant differences in the mean number of fruit and vegetable portions consumed by the general population at each time point (T1 3.8 ± 2.6 vs T2 3.7 ± 2.5 vs T4 3.9 ± 2.3 portions, p > 0.05). Members consumed more portions of fruit and vegetables each day than the general population at all time points (all p < 0.001).

The proportion of members consuming the UK Government’s recommendation of five portions of fruit and vegetables a day increased after joining (83.9% vs 46.0%; T1 vs T0, p < 0.001) and remained consistent at T2 (82.2%, p > 0.05) and T4 (79.9%, p > 0.05). At all time points, more members were reaching the recommendation compared to the general population (all p < 0.001).

Sugary Drink Consumption

From the scaled Likert responses, members consumed sugary drinks less frequently after joining (1.1 ± 0.6 vs 1.4 ± 1.0; T1 vs T0, p < 0.001) and less frequently than the general population at all time points (T1 1.1 ± 0.6 vs 1.8 ± 1.2; T2 1.1 ± 0.5 vs 1.7 ± 1.1; T4 1.2 ± 0.7 vs 1.7 ± 1.1; all p < 0.001).

Wholegrain Consumption

Wholegrain consumption changed over time for members (F(2.78, 480.99) = 11.90, p < 0.001). From the scaled Likert responses, compared to T0 (2.4 ± 1.2), members consumed wholegrains more frequently at T1 (2.6 ± 1.1), T2 (2.9 ± 1.1), and T4 (2.9 ± 1.2; all p < 0.001). Members also consumed wholegrains more frequently than the general population at all time points (T1 2.6 ± 1.1 vs 2.4 ± 1.1; T2 2.9 ± 1.1 vs 2.5 ± 1.2; T4 2.9 ± 1.2 vs 2.6 ± 1.2; all p < 0.01).

Diet Quality

**Figure 3:**
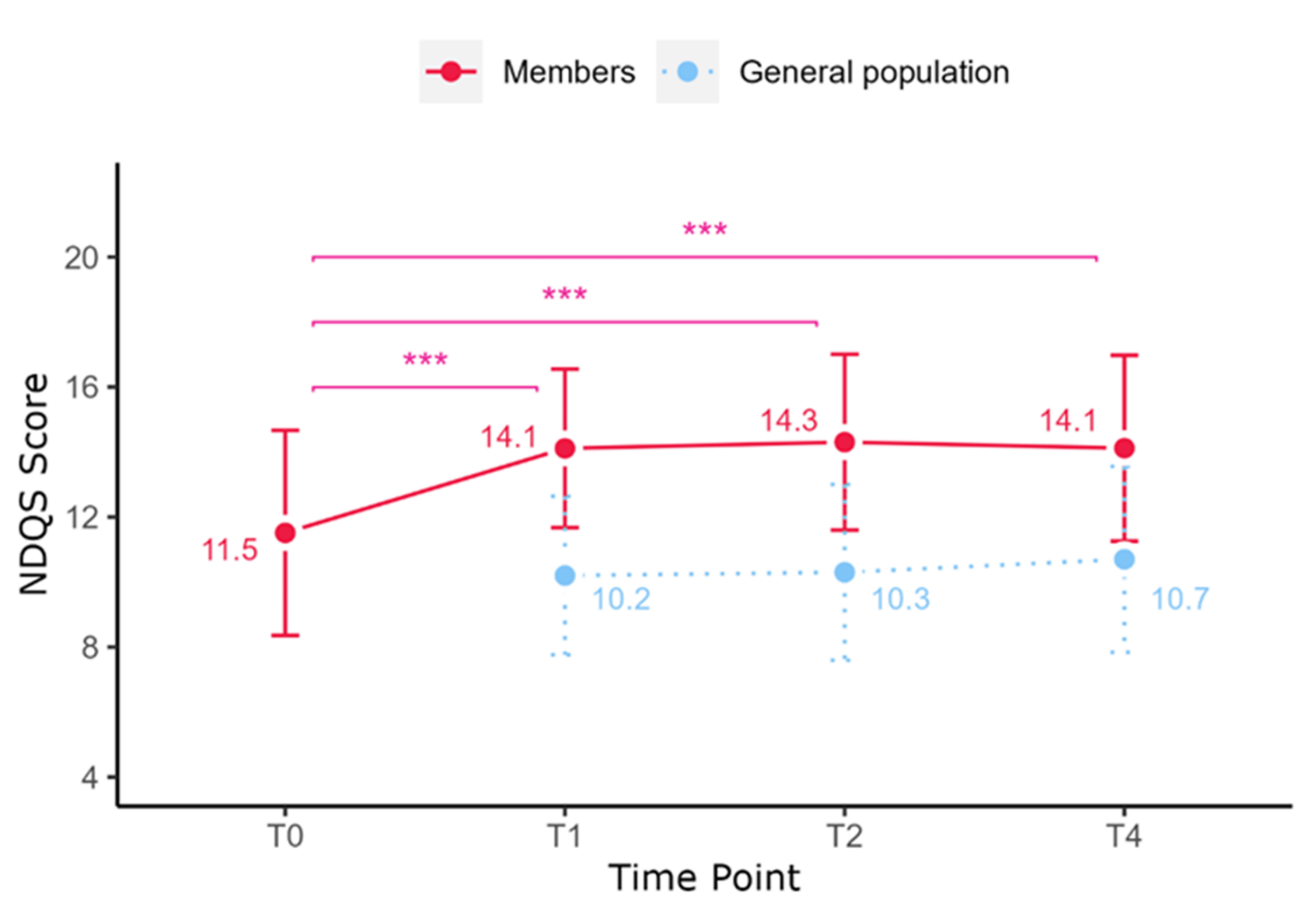
Changes in NDQS scores over time. *p < 0.05, **p < 0.01, ***p < 0.001. NDQS: Nutrient-based Diet Quality Score.

For Slimming World members, NDQS scores changed over time (F(2.67, 462.54) = 80.36, p < 0.001) (Table 2). Scores increased after joining (14.1 ± 2.4 vs 11.5 ± 3.2; T1 vs T0, p < 0.001) and were sustained at T2 (14.3 ± 2.7) and T4 (14.1 ± 2.9) (both p > 0.05). Members had higher diet quality scores compared to the general population at T1, T2, and T4 (all p < 0.001; Figure 3).

**Table 2:** Mean values ± 95% confidence intervals for diet quality, physical activity, sedentary behaviour, and well-being at each time point. SW: Slimming World; Ref group: General population reference group; T0: Before joining Slimming World; T1: Up to 4 weeks after joining Slimming World; T2: 3-month data; T3: 6-month data; T4: 12-month data.

	T0	T1	T2	T4
Diet quality score (95% CI)				
SW	11.5 (11.0, 12.0)	14.1 (13.7, 14.5)	14.3 (13.9, 14.7)	14.1 (13.7, 14.6)
Ref group	-	10.2 (10.1, 10.4)	10.3 (10.2, 10.5)	10.7 (10.5, 10.9)
Physical activity (hours/week) (95% CI)				
SW	-	4.6 (3.5, 5.7)	6.7 (4.9, 8.5)	6.5 (5.5, 7.6)
Ref group	-	5.4 (4.8, 6.0)	5.2 (4.2, 4.8)	5.2 (4.3, 6.1)
Sedentary behaviour (hours/week) (95% CI)				
SW	-	36.4 (33.7, 39.1)	33.5 (30.8, 36.2)	32.4 (29.4, 35.4)
Ref group	-	36.8 (35.6, 38.0)	38.4 (37.3, 39.5)	39.2 (37.8, 40.8)
Well-being score (95% CI)				
SW	-	27.9 (27.0, 28.8)	29.4 (28.4, 30.4)	27.4 (26.4, 28.4)
Ref group	-	22.7 (22.6, 22.8)	24.6 (24.2, 25.0)	24.5 (24.0, 25.0)

Dietary behaviours

Consumption of Sugary Foods

The frequency at which members consumed sugary foods changed over time (F(2.8, 462.8) = 117.2, p < 0.001). From the scaled Likert responses, members consumed sugary foods less frequently soon after joining (1.9 ± 1.2 vs 4.0 ± 1.3; T1 vs T0, p < 0.0001). The frequency of sugary food consumption increased at T2 (1.9 ± 0.9 vs 2.5 ± 0.1; T1 vs T2, p < 0.0001) and remained consistent at T4 (2.7 ± 0.1, p > 0.05). Compared to before joining, members consumed sugary foods less frequently at all time points after joining (all p < 0.001). For the general population, the frequency of sugary food intake remained consistent over time (F(2, 3696) = 0.36, p > 0.05). The general population consumed sugary foods more frequently than members at T1 (3.4 ± 1.2 vs 1.9 ± 0.9), T2 (3.4 ± 1.2 vs 2.5 ± 0.1), and T4 (3.4 ± 1.2 vs 2.7 ± 0.1) (all p < 0.001).

Consumption of Fatty Foods

The frequency of members’ fatty food consumption changed over time (F(2.8, 462.3) = 173.9, p < 0.001). The scaled Likert responses showed consumption dropped after joining (1.4 ± 0.7 vs 3.4 ± 1.2; T1 vs T0, p < 0.001), increased from T1 to T2 (1.4 ± 0.7 vs 1.6 ± 1.0; T1 vs T2, p < 0.05), and then remained consistent between T2 and T4 (1.6 ± 1.0 vs 1.8 ± 1.2; T2 vs T4, p > 0.05). Members consumed fatty foods less frequently at all time points compared to before joining.

The frequency of fatty food intake for the general population remained consistent over time (F(2, 3696) = 6.8, p > 0.05). Members consumed fatty foods less frequently than the general population at T1 (1.4 ± 0.7 vs 3.0 ± 1.1), T2 (1.6 ± 1.0 vs 2.9 ± 1.1), and T4 (1.8 ± 1.2 vs 2.9 ± 1.1) (all p < 0.001).

Alcohol Intake

Overall, a change in alcohol consumption was observed for members over time (F(2.4, 230.6) = 5.33, p < 0.05). At T2, members were consuming fewer units of alcohol compared to T0 (11.0 ± 14.8 vs 16.7 ± 17.1 units/week, p < 0.05). Between T2 and T4, weekly alcohol intake increased slightly, but remained below pre-joining levels (12.7 ± 13.4 vs 16.7 ± 17.1 units/week; T4 vs T0, p < 0.001). Compared to the general population, members consumed fewer units of alcohol per week at T1 (13.5 ± 14.9 vs 16.2 ± 26.2 units/week, p < 0.01) and T2 (11.0 ± 14.8 vs 14.3 ± 19.9 units/week, p < 0.001).

Before joining, 41.2% of members consumed alcohol within the UK Government recommendation of <14 units per week. The proportion of members meeting this recommendation increased at T1 (78.4%), T2 (81.4%), and T4 (76.3%) (all p < 0.001). At all time points after joining, a greater proportion of members consumed alcohol within UK Government recommendations than the general population (T1: 78.4% vs 63.0%; T2: 81.4% vs 68.0%; T4: 76.3% vs 63.4%; all p < 0.05).

Changes in physical activity

Moderate-intensity Activity

The number of hours of moderate-intensity physical activity undertaken by members increased between T1 and T2 (4.6 ± 7.4 vs 6.7 ± 10.1 hours/week, p < 0.01) (Table 2). Members maintained their activity level at T4 and participated in more hours of activity compared to T1 (6.5 ± 6.0 vs 4.6 ± 7.4 hours/week, p < 0.01).

**Figure 4:**
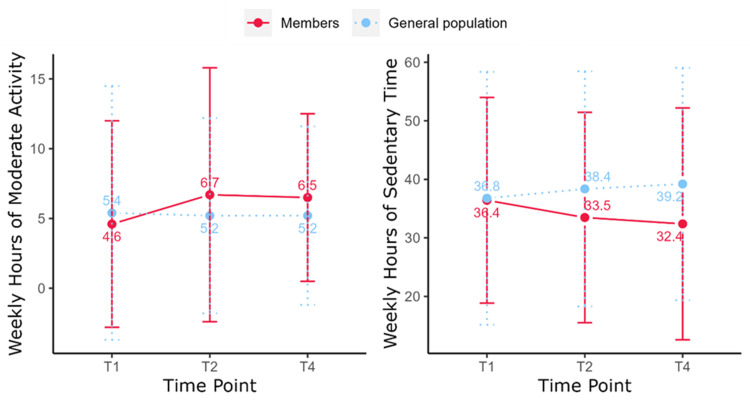
Weekly hours of moderate-intensity physical activity and sedentary time among Slimming World (SW) members and the general population reference group.

The number of hours of moderate-intensity physical activity reported by the general population at each time point remained consistent (5.4 ± 10.1 vs 5.2 ± 7.0 vs 5.2 ± 6.4 hours/week; T1 vs T2 vs T4, p > 0.05). At T1, members participated in fewer hours of physical activity per week compared to the general population (4.6 ± 7.4 vs 5.4 ± 10.1 hours/week, p < 0.001). At T2, members spent more time being active (6.7 ± 10.1 vs 5.2 ± 7.0 hours/week, p < 0.001), and this remained the case at T4 (6.5 ± 6.0 vs 5.2 ± 7.0 hours/week, p < 0.001) (Figure 4).

Physically Active Classification

The proportion of members classified as active according to Sport England’s definition did not change significantly over time (p > 0.05). However, a greater proportion of members were doing sufficient physical activity to be classified as active at T4 compared with T1 and T2 (78.8% vs 66.4% vs 65.9%; T4 vs T1 vs T2).

At T1, fewer members were considered active compared to the general population, but by T4, a greater proportion of members were engaging in sufficient activity to be classified as active. However, these differences were not statistically significant at any time point (p > 0.05).

Sedentary Time

Over the 12 months, there was a slight decrease in the time members reported being sedentary, but the changes were not significant (p > 0.05) (Table 2). At T1, members’ sedentary hours per week were similar to those of the general population (36.4 ± 17.6 vs 36.8 ± 21.6 hours, p > 0.05). By T2, members spent less time sedentary compared to the general population (33.5 ± 18.0 vs 38.4 ± 20.1 hours, p < 0.01), and this difference increased further at T4 (32.4 ± 19.8 vs 39.2 ± 19.2 hours, p < 0.001) (Figure 4).

Mental well-being

Mental well-being scores varied over time for members (F(2, 346) = 12.14, p < 0.001), improving from T1 to T2 (27.9 ± 6.03 vs 29.4 ± 6.44, p < 0.05) and decreasing between T2 and T4 (29.4 ± 6.44 vs 27.4 ± 6.86, p < 0.05), with no difference between T1 and T4 (Table 2).

Mental well-being scores also changed over time for the general population (F(2, 3696) = 46.15, p < 0.001), increasing from T1 to T2 (22.7 ± 3.93 vs 24.6 ± 7.52, p < 0.001) with no difference between T2 and T4 (24.6 ± 7.52 vs 24.5 ± 7.75, p > 0.05).

**Figure 5:**
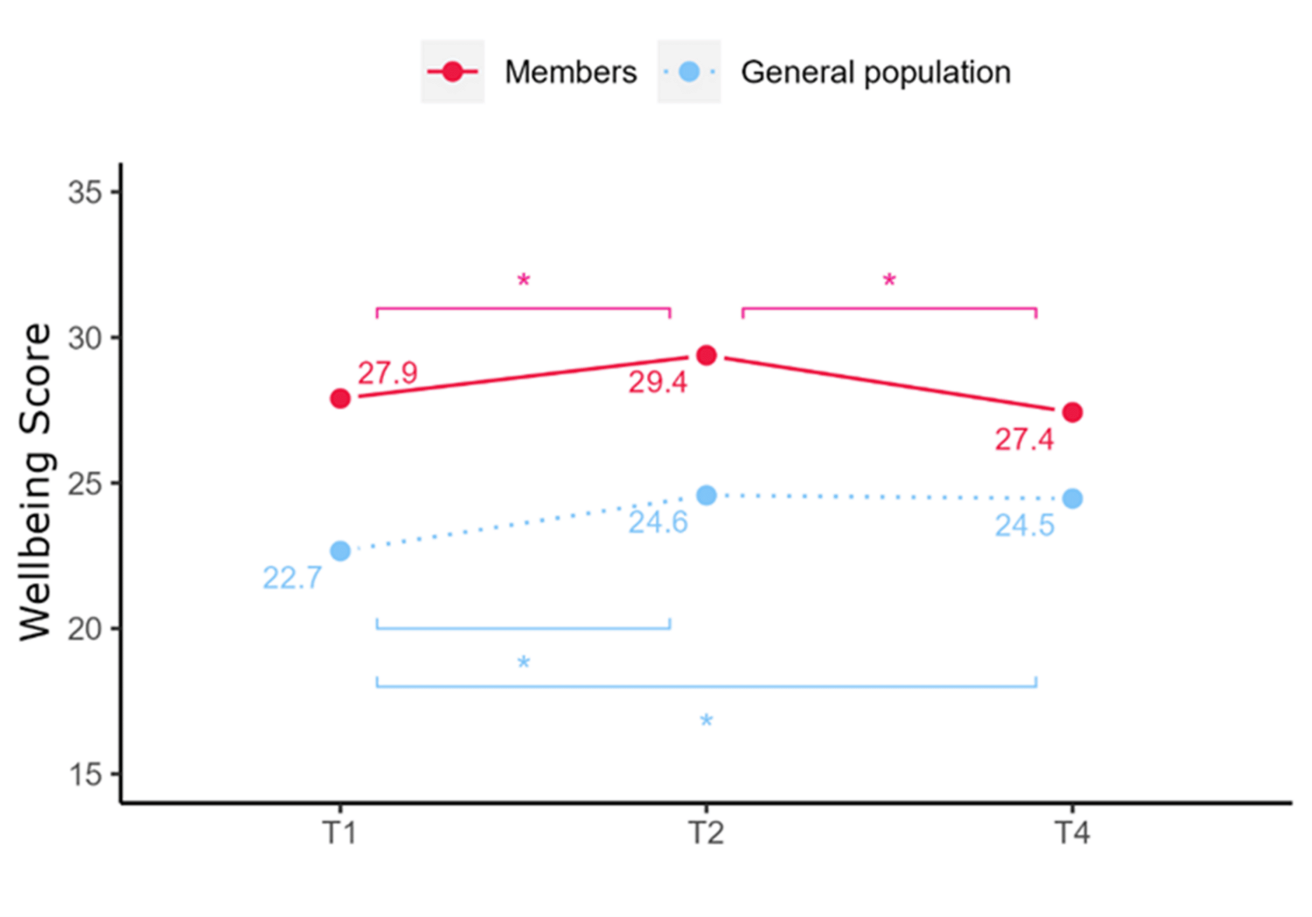
Changes in well-being scores among Slimming World (SW) respondents and the reference group at each time point.

At each time point, members had significantly higher mental well-being scores than the general population (Figure 5).

Wider influence of Slimming World membership

At T2, among those living with a partner (n = 122, 70.1%), 79.5% reported that they had influenced their partner to eat healthier meals due to their Slimming World membership. Of the members living with children (n = 47, 27.0%), 66.0% reported influencing their children to eat healthier meals.

The positive influence on partners’ and children’s eating habits observed at T2 remained stable at T4 (partner = 71.3%; child/children = 57.9%; both p > 0.05). At T2, 40.7% of members reported encouraging other household members to become more active, and this proportion remained consistent at T4 (40.5%, p > 0.05).

## Discussion

The aim of the current study was to investigate changes in health-related behaviours and well-being of SW members over a 12-month period after first joining the weight management programme. Data on newly joining members were compared to cross-sections of the general population who had a mean BMI in the obese category but were not actively managing their weight with a commercial organisation at the time of survey completion. This method offered a pragmatic approach to including a pseudo-control group for a non-randomised intervention study conducted in a real-life setting.

The 174 members who completed all three surveys were predominantly female and had a mean starting BMI within the obese category, according to the WHO classification [15]. At the 12-month time point, these members had lost 7.5% of their starting body weight, equating to a mean BMI reduction of 2.6 kg/m². Similar clinically beneficial weight outcomes have been observed in previous large-scale studies. For example, a service evaluation of over one million self-funding adults attending the community weight management programme found a mean weight loss of 6.0% at 12 months [7], whilst an evaluation of adults referred found a mean weight loss of 7.3% at 24 months [16]. A retrospective, real-world U.S. cohort study of 7,881 adults using electronic health record data to identify people with overweight or obesity without type 2 diabetes who initiated injectable semaglutide or tirzepatide found a mean weight reduction at one year of 8.7% (SD 9.6%) [17]. While the mean age was similar to the participants in our study, the gender ratio was different, with an approximately 75:25 (female:male) ratio, which may have contributed to the lower level of compliance with the injectables.

Data from this study showed significant changes in dietary behaviours soon after joining the programme, many of which were maintained over the 12-month study period, with members maintaining a healthier diet than the comparator group. Changes included eating more fruits and vegetables, consuming wholegrains more frequently, consuming fatty and sugary foods/drinks less frequently, and reducing alcohol intake in line with recommendations. More members were meeting the recommended five-a-day despite having similar intakes at baseline. The increase in the nutrient-based diet quality score, alongside the mean weight loss, suggests that members were supported to adopt healthier eating behaviours that reduced the energy density of their diet. This builds on previous observational research that found members are supported to eat a healthy, balanced diet while maintaining the energy deficit required for sustainable weight loss [18]. Members’ pre-joining dietary behaviours were similar to those of the comparator group, as well as to national datasets in the UK [19], suggesting that improvements in members’ diets were due to the healthy eating advice encouraged.

A greater proportion of members were doing sufficient physical activity to be classified as active at 12 months, which, alongside diet-related behaviour changes, was likely to have contributed to the weight loss observed [20]. Reducing sedentary behaviour and physical inactivity is increasingly recognised as having metabolic health benefits [21], and members reduced their sedentary behaviour, spending less time being inactive compared to the general population.

While members’ well-being scores varied slightly over time, potentially influenced by COVID-19 and the social restrictions imposed at the time of the 12-month survey (T4), they had significantly higher mental well-being scores than the general population at all time points after joining. This may be due to the peer support and social connections developed as part of a community weight management programme [22]. The findings also suggest wider benefits of the support, with more than three-quarters of members influencing their partner to eat healthier meals and two-thirds positively influencing their children. Despite a more limited influence on activity levels, there was still a positive impact, with nearly half of the members encouraging their partner and children to become more active.

A recent qualitative review systematically investigating the barriers and facilitators to diet and physical activity recognised the difficulties that adults can have in maintaining changes and meeting recommendations for a healthy diet and physical activity levels [23]. The findings from that review suggest that interventions should focus on helping participants find ways to easily incorporate guidelines into their routines as feasible habits. Social support and accountability were also found to be important facilitators, alongside inclusive spaces where people do not feel stigmatised. This study suggests that a community-based weight management programme such as SW can offer these facilitators in a scalable way to support adults and their families in making positive dietary and activity changes that improve their general and psychological well-being.

Whilst this study has highlighted the positive changes in health-related behaviours following weight management support from the intervention, further research is warranted to understand potential response biases within the data. As the response rate for the survey at the 12-month time point was low, with 174 (9.2%) of the original baseline sample completing surveys at both the 3- and 12-month time points, this sample may not be fully representative of all adults joining the programme. However, while the study response could be considered low, it aligns with other longer-term pragmatic studies of people living with obesity [9, 24].

The majority of respondents were female, and thus the same findings may not be observed if more males had participated. In addition, despite attempts to match members and the general population for key demographics, members were, on average, older and had a higher mean BMI than the general population. Therefore, observed differences between the groups could be attributed in part to demographic differences, and results should be interpreted with this in mind. We have not reported whether participants responding to the survey had any weight-related chronic disease, but it is likely that a proportion did have associated health conditions. A further limitation is that the use of self-reported data may have led to bias in the reporting of weight, as well as diet and lifestyle behaviours. However, this is a common limitation wherever self-reported data are utilised, and any bias is likely to be present in both the member and reference groups.

## Conclusions

Consuming a balanced diet and engaging in regular physical activity have many health benefits. However, many adults find it difficult to adhere to dietary and activity recommendations, particularly within weight management interventions, and not all weight loss approaches encourage healthier eating and exercise behaviours.

This study found that SW members who completed the survey at all three time points achieved and maintained a clinically significant mean weight loss 12 months after joining. They also reported positive changes across numerous health-related behaviours, including increased fruit and vegetable intake, improved overall diet quality, and higher levels of physical activity, alongside reduced sedentary time. Many of these changes were observed early in their weight management journey and were sustained at 12 months, including improvements in self-reported physical health and greater levels of mental well-being compared to the reference group.

The self-reported data from those responding to the survey at 12 months suggest that the programme can be effective in supporting adults living with overweight and obesity to adopt healthier behaviours for long-term weight management, with or without the need for weight loss medication. The weight management support may also have wider benefits, with many respondents indicating that their families adopted healthier dietary habits.

## Appendices

Appendix 1

Health and Well-being Survey - 1b: Member 3-Month Follow-Up

Participant Information - Health and Well-being Survey

Information for Participants

Please read this information carefully and make sure you’re happy with everything before agreeing to take part.

What is the purpose of this study?

The aim of this online survey is to collect information on the health, well-being, and lifestyle behaviours of Slimming World members, and how these change over time.

Who is being asked to take part?

We’re inviting all new Slimming World members who have joined within the past two weeks to take part in this initial survey.

What does the study involve?

The online survey will take around 20 minutes to complete. It will ask you a bit about yourself, including questions about your health, well-being, physical activity, and diet. We’ll be inviting you to take part in four surveys (this one plus three more) over the course of a year. For each of the four surveys that you complete, we’ll enter you into a prize draw for a chance to win high street shopping vouchers.

We’d also love to be able to contact you about your diet and lifestyle after the four surveys have been completed, for long-term follow-up research.

Do I have to take part?

Your participation is entirely voluntary, and you do not have to take part if you do not wish to.

Are there any benefits or risks to taking part in the research?

By participating in this study, you’ll be helping Slimming World to further develop its programme and support members in the long term. There are no risks associated with taking part.

Are there any costs or incentives to taking part in the research?

There are no costs to taking part in the survey. For each survey, there will be an opportunity to opt in to a prize draw for the chance to win a high street shopping voucher.

The prize draws are as follows:

Completion of the first survey: a chance to win one of five £50 shopping vouchers.

Completion of the second survey: a chance to win either one £250, one £100, or one of three £50 vouchers.

Completion of the third survey: a chance to win either one £250, one £100, or one of three £50 vouchers.

Completion of the fourth survey: a chance to win either one £250, one £100, or one of three £50 vouchers.

To be entered into the prize draw, you’ll need your Slimming World membership number (if you’re a group member) and your email address. These will only be used to invite you to each survey and to enter you into the prize draw.

What happens to the collected information?

All data will be stored in a password-protected file within a secure network. The data will be anonymised, and contact details such as email addresses will be stored separately and securely. No identifiable information will be used in reporting.

What will happen to the results of this study?

The results of the study will be reported as part of a Slimming World research project to help the company further develop its service to members. The results may also be submitted for publication in a scientific journal or presented at a scientific conference. However, no identifiable data will be used in any reporting.

You can contact the Nutrition, Research and Health team to request a summary of the results when the study is finished (see contact details below).

Privacy information for research participants

For information about the University’s obligations with respect to your data and who you can contact, please visit: 🔗 https://www.nottingham.ac.uk/utilities/privacy.aspx

For information about Slimming World’s obligations with respect to your data and who you can contact, please visit: 🔗 https://www.slimmingworld.co.uk/privacy-policy

Why we collect your personal data

We collect personal data under the terms of the University’s Royal Charter in our capacity as a teaching and research body to advance education and learning. Any personal data collected will only be used for research purposes and will remain completely confidential.

Legal basis for processing your personal data under GDPR

The legal basis for processing your personal data on this occasion is Article 6(1a) - consent of the data subject.

In addition to this legal basis, the University must meet a further condition when processing any special category data, including personal data revealing racial or ethnic origin, political opinions, religious or philosophical beliefs, trade union membership, genetic or biometric data, or data concerning health, sex life, or sexual orientation.

The basis for processing your sensitive personal data on this occasion is Article 9(2a) - the data subject has given explicit consent to the processing.

How long we keep your data

The University of Nottingham and Slimming World may store your data for up to 25 years, and for a period of no less than 7 years after the research project finishes. The researchers who gathered or processed the data may also store the data indefinitely and reuse it in future research that has ethical approval. Any identifiable data will be removed before it is made available to other researchers.

Complaints procedure

If you have any concerns about the research, please contact the supervisors of the project using the contact details provided below. If this does not resolve the matter to your satisfaction, you may contact the University of Nottingham’s Research Ethics Officer.

Withdrawal from the study

You can withdraw from the study at any point without needing to provide a reason. There will be no negative consequences as a result of withdrawal. Any information you have provided will be deleted upon withdrawal.

Who has reviewed the study?

The study has been reviewed and approved by the School of Biosciences Research Ethics Committee.

Thank you for considering taking part in this research.

Who can I contact if I have any questions?

Slimming World Nutrition, Research and Health Team Email: nutrition.research@slimmingworld.co.uk

Dr Amanda Avery Contact No.: +44 115 951 6238 Email: amanda.avery@nottingham.ac.uk

If you’re happy to continue, please click the arrow to proceed to the consent form.

End of Block: Participant Information - Lifestyle

Start of Block: Consent Form

Consent Statement

I confirm that:

I have read the Participant Information Sheet, and the nature and purpose of the research project have been explained to me.

I have had the opportunity to ask questions.

I understand the purpose of the research project and my involvement in it.

I understand that my participation is voluntary and that I may withdraw from the research project at any stage without having to give any reason, and that withdrawing will not penalise or disadvantage me in any way.

I understand that while information gained during the study may be published, any information I provide will remain confidential, and no information that could lead to the identification of any individual will be disclosed in any reports on the project or to any other party.

No identifiable personal data will be published.

I understand that data will be securely stored.

I understand that the information provided may be used in other research projects that have ethical approval, but that my name and contact information will be removed before it is made available to other researchers.

I understand that I may contact the researcher if I require further information about the research and that I may contact the Research Ethics Officer of the School of Biosciences, University of Nottingham, if I wish to make a complaint relating to my involvement in the research.

I understand that I may be contacted by email after the four surveys have been completed for long-term follow-up research, but that participation in this is entirely my choice.

I agree to take part in the above research project. By selecting “Yes”, I agree to all of the above terms and conditions and agree to participate in this research project.

○ Yes  ○ No

Start of Block: Contact Information

Contact Information

Are you still a member of Slimming World? ○ Yes  ○ No

If “Yes”:

Since you completed the previous survey a few months ago, have you been a member the whole time, or did you have a break and re-join? ○ I have been a Slimming World member since I completed the previous survey ○ I had a break from Slimming World for a while, then re-joined ○ Not sure / can’t remember

Please enter the email address you registered with (used only for the prize draw and to send the next survey in 3 months): [Email address ________________________________________________]

Are you an online or group member? ○ Online member  ○ Group member

If “Group member”: Please enter your membership (card) number (found on the front of your card): [Membership number ___________________________________________]

End of Block: Contact Information

Start of Block: Demographics

Demographics

What is your gender? ○ Female ○ Male ○ Transgender Female ○ Transgender Male ○ Non-binary ○ Other: ____________________________________ ○ Prefer not to answer

Please enter your month and year of birth: Month (e.g., May): ____________________________________ Year (e.g., 1970): ______________________________________

End of Block: Demographics

Start of Block: Census

Census

What country do you live in? ○ England ○ Wales ○ Scotland ○ Northern Ireland ○ Republic of Ireland ○ Other (please specify): _______________________

If country ≠ Republic of Ireland: Please enter your postcode: [Postcode _________________________________________________]

Ethnic origin (country-specific lists):

If England: ○ English/Welsh/Scottish/Northern Irish/British ○ Irish ○ Gypsy or Irish Traveller ○ Any other White background (please describe): ________________________ ○ White and Black Caribbean ○ White and Black African ○ White and Asian ○ Any other Mixed/Multiple background (please specify): ________________ ○ Indian ○ Pakistani ○ Bangladeshi ○ Chinese ○ Any other Asian background (please describe): ________________________ ○ Caribbean ○ African ○ Any other Black background (please describe): ________________________ ○ Arab ○ Any other ethnic group (please describe): ____________________________

If Wales: (wording adjusted for Wales) ○ Welsh/English/Scottish/Northern Irish/British … (same structure as above)

If Scotland: (Scotland categories) ○ Scottish ○ Other British ○ Irish ○ Gypsy/Traveller ○ Polish ○ Any other White ethnic groups (please describe): ______________________ ○ Any Mixed or Multiple ethnic groups (please describe): ________________ ○ Pakistani, Pakistani Scottish or Pakistani British ○ Indian, Indian Scottish or Indian British ○ Bangladeshi, Bangladeshi Scottish or Bangladeshi British ○ Chinese, Chinese Scottish or Chinese British ○ Any other Asian (please describe): __________________________________ ○ African, African Scottish or African British ○ Any other African (please describe): _________________________________ ○ Caribbean, Caribbean Scottish or Caribbean British ○ Black, Black Scottish or Black British ○ Any other Caribbean or Black (please describe): ______________________ ○ Arab, Arab Scottish or Arab British ○ Any other ethnic group (please describe): ____________________________

If Northern Ireland: ○ White ○ Irish Traveller ○ White and Black Caribbean ○ White and Black African ○ White and Asian ○ Any other Mixed/Multiple ethnic background (please describe): _________ ○ Indian ○ Pakistani ○ Bangladeshi ○ Chinese ○ Any other Asian background (please describe): ________________________ ○ African ○ Caribbean ○ Any other Black/African/Caribbean background (please describe): ______ ○ Arab ○ Any other ethnic group (please describe): ____________________________

If Republic of Ireland: ○ Irish ○ Irish Traveller ○ Any other White background ○ African ○ Any other Black background ○ Chinese ○ Any other Asian background ○ Other (please describe): ___________________________________________

Household income (choose the relevant currency list):

If country ≠ Republic of Ireland: ○ Under £10,000 ○ £10,000-£14,999 ○ £15,000-£19,999 ○ £20,000-£24,999 ○ £25,000-£29,999 ○ £30,000-£39,000 ○ £40,000-£49,999 ○ Over £50,000 ○ Prefer not to say

If Republic of Ireland: ○ Under €10,000 ○ €10,000-€14,999 ○ €15,000-€19,999 ○ €20,000-€24,999 ○ €25,000-€29,999 ○ €30,000-€39,000 ○ €40,000-€49,999 ○ Over €50,000 ○ Prefer not to say

End of Block: Census

Start of Block: Health 1

Health 1

Please tell us your height. I would like to enter my height in… ○ Centimetres ○ Feet and inches

If “Centimetres”: Height (cm): ______________________________________________

If “Feet and inches”: Feet: ____________________ Inches: ________________________

Please tell us your current weight. I would like to enter my weight in… ○ Stones and pounds ○ Kilograms

If “Stones and pounds”: Stones: ____________________ Pounds: ______________________

If “Kilograms”: Kilograms: ________________________________________________

Are you currently pregnant? ○ Yes ○ No

Are you currently breastfeeding? ○ Yes ○ No

Which option best describes your usual eating habits? ○ Vegan (no dairy, eggs or any animal products) ○ Vegetarian (no meat, poultry, game, fish or shellfish) ○ Pescatarian (eat fish, but not meat or poultry) ○ Meat eater (eat meat and/or poultry) ○ Flexitarian (vegetarian-only days plus mixed diet other days) ○ Other: _________________________ 

○ Don’t know

End of Block: Health 1

Start of Block: Health 2

These next few questions ask for your views about your health.  This information will help keep track of how you feel and how well you are able to do your usual activities.  Answer each question by choosing just one answer.  If you are unsure how to answer a question, please give the best answer you can.

Health 2

(Choose one answer for each question.)

In general, would you say your health is… ○ Excellent ○ Very good ○ Good ○ Fair ○ Poor

Compared to one year ago, how would you rate your health now? ○ Much better ○ Somewhat better ○ About the same ○ Somewhat worse ○ Much worse

End of Block: Health 2

Start of Block: Health 2

These next few questions ask for your views about your health.  This information will help keep track of how you feel and how well you are able to do your usual activities.  Answer each question by choosing just one answer.  If you are unsure how to answer a question, please give the best answer you can.

Health 2

(Choose one answer for each question.)

In general, would you say your health is… ○ Excellent ○ Very good ○ Good ○ Fair ○ Poor

Compared to one year ago, how would you rate your health now? ○ Much better ○ Somewhat better ○ About the same ○ Somewhat worse ○ Much worse

End of Block: Health 2

Start of Block: Health Conditions

Please indicate whether you have any of the following conditions or health problems (tick all that apply): ☐ Anxiety                     ☐ Breathlessness ☐ Cardiovascular disease             ☐ Chronic back pain ☐ Depression                  ☐ Dyslipidaemia (raised blood lipids) ☐ Joint pain                   ☐ Osteoarthritis ☐ Polycystic ovary syndrome (PCOS)       ☐ Poor mobility ☐ Raised blood pressure            ☐ Raised cholesterol ☐ Sleep apnoea                 ☐ Type 1 diabetes ☐ Pre-diabetes                 ☐ Type 2 diabetes ☐ Other (please specify): ___________________________________________

Since you joined Slimming World, has your doctor or healthcare team advised that you change the dose of any prescribed medication as a result of your weight loss? Please tick all that apply.

Stopped

Reduced

Not changed

Increased

N/A

Diabetes medication

o  

o  

o  

o  

o  

Cholesterol medication

o  

o  

o  

o  

o  

Blood pressure medication

o  

o  

o  

o  

o  

Weight loss medication

o  

o  

o  

o  

o  

Depression medication

o  

o  

o  

o  

o  

Anxiety medication

o  

o  

o  

o  

o  

Polycystic ovary syndrome (PCOS) medication

o  

o  

o  

o  

o  

Mobility medication

o  

o  

o  

o  

o  

Fertility medication

o  

o  

o  

o  

o  

If you have any other comments regarding changes in prescribed medication, please type these below.

________________________________________________________________

________________________________________________________________

________________________________________________________________

________________________________________________________________

End of Block: Health conditions

Start of Block: Mood

This next question is about how you have been feeling during the past 4 weeks.  For each question, please give the one answer that comes closest to the way you have been feeling.

How much of the time during the past 4 weeks...

All of the time

Most of the time

A good bit of the time

Some of the time

A little of the time

None of the time

Have you felt calm and peaceful?

o  

o  

o  

o  

o  

o  

Did you have a lot of energy?

o  

o  

o  

o  

o  

o  

Have you felt downhearted and low?

o  

o  

o  

o  

o  

o  

Have you been a happy person?

o  

o  

o  

o  

o  

o  

Have you felt in a sociable mood?

o  

o  

o  

o  

o  

o  

Have you felt stressed?

o  

o  

o  

o  

o  

o  

Have you felt anxious?

o  

o  

o  

o  

o  

o  

End of Block: Mood

Start of Block: Smoking

Have you ever smoked regularly (not including electronic cigarettes)? ○ Yes ○ No

Display This Question: If Have you ever smoked regularly (not including electronic cigarettes)? = Yes

Do you smoke at all nowadays (not including electronic cigarettes)? ○ Yes ○ No

Display This Question: If Have you ever smoked regularly (not including electronic cigarettes)? = No

Was your decision to stop smoking influenced by Slimming World in any way? ○ Yes ○ No ○ Not sure

End of Block: Smoking

Start of Block: Diet 1

How many portions of fruit did you eat yesterday? Please include all fruit - fresh, frozen, dried, or tinned - as well as stewed fruit, fruit juices, and smoothies. Please note: fruit juice and smoothies count as one portion only, no matter how much you drink.

How many portions of vegetables did you eat yesterday? Please include fresh, frozen, raw, or tinned vegetables, but do not include any potatoes. Please note: beans and pulses count as one portion only, no matter how much you eat.

In a typical week, how often do you do the following?

Behaviour More than once a day Daily 4-6 times a week 1-3 times a week  Never/Occasionally

Drink sugary drinks (not low-calorie or diet)○○○○○

Eat wholegrains such as wholegrain or wholemeal versions of pasta, cereals, and bread○○○○○

End of Block: Diet 1

Start of Block: Diet 2

In a typical month, how often do you do the following?

BehaviourOnce a day or moreMore than once a week but less than dailyAbout once a weekMore than once a month but less than once a weekOnce a month or less

Eat takeaways or fast food○○○○○

Cook a meal from scratch○○○○○

Eat out (e.g., in restaurants or pubs)○○○○○

Eat in front of the television○○○○○

Eat sitting at a table○○○○○

Eat fatty foods (e.g., burgers, sausages, pizza, pies, crisps, and chips that are not made using Slimming World recipes)○○○○○

Eat sugary foods (e.g., sweets, cakes, pastries, and chocolate)○○○○○

End of Block: Diet 2

Start of Block: Diet 3

How much do you agree or disagree with the following statements about cooking and eating out?

Statement Strongly agree Agree a little Neither agree nor disagree Disagree a little Strongly disagree

I struggle to find time to cook.○○○○○

I enjoy cooking from scratch.○○○○○

Cooking from scratch is expensive.○○○○○

Eating out is difficult when trying to manage my weight.○○○○○

I avoid eating out when I am trying to manage my weight.○○○○○

End of Block: Diet 3

Start of Block: Activities

Are your day-to-day activities limited because of a health problem or disability that has lasted, or is expected to last, at least 12 months? Please include problems related to ageing. ○ Yes, limited a lot ○ Yes, limited a little ○ No

In the past seven days, have you done any of these activities? (These could be done with aids such as a walking stick, wheelchair, walker, swimming aid, hand bike, or similar.)

▢ A continuous walk lasting at least 10 minutes ▢ A cycle ride ▢ A sport, swimming, or fitness activity such as fitness classes or dance ▢ A strength exercise or activity such as yoga, Pilates, resistance bands, weights, squats, or sit-ups ▢ ⊗ None of these

End of Block: Activities

Start of Block: Walking

Display This Question: If In the past 7 days, have you done any of these activities? = A continuous walk lasting at least 10 minutes

Question 1: In the past 7 days, on how many days did you do a walk lasting at least 10 minutes? This could include using aids such as a walking stick, wheelchair, or walker. ○ Days ________________________________________________

Question 2: On average, how much time did you spend walking on each day that you did this activity? ○ Minutes per day (e.g., 1.5 hours = 90 minutes) ________________________________________________

Question 3: Was the effort you put into walking usually enough to raise your breathing rate? ○ Yes ○ No

End of Block: Walking

Start of Block: Cycling

Display This Question: If In the past 7 days, have you done any of these activities? = A cycle ride

Question 1: In the past 7 days, on how many days did you do a cycle ride? This could include using a hand bike or similar equipment. ○ Days ________________________________________________

Question 2: On average, how much time did you spend cycling on each day that you did this activity? ○ Minutes per day (e.g., 1.5 hours = 90 minutes) ________________________________________________

Question 3: Was the effort you put into cycling usually enough to raise your breathing rate? ○ Yes ○ No

End of Block: Cycling

Start of Block: Sports / Swimming / Fitness

Display This Question: If In the past 7 days, have you done any of these activities? = A sport, swimming, or fitness activity (such as fitness classes or dance)

Question 1: In the past 7 days, on how many days did you do a sport, swimming, or fitness activity (such as fitness classes or dance)? This could include using aids that help you move, such as a wheelchair, swimming aids, or similar support. ○ Days ________________________________________________

Question 2: On average, how much time did you spend doing sport, swimming, or fitness activities (such as fitness classes or dance) on each day that you did the activity? ○ Minutes per day (e.g., 1.5 hours = 90 minutes) ________________________________________________

Question 3: Was the effort you put into doing sport, swimming, or fitness activities (such as fitness classes or dance) usually enough to raise your breathing rate? ○ Yes ○ No

End of Block: Sports / Swimming / Fitness

Start of Block: Strength

Display This Question: If In the past 7 days, have you done any of these activities? = A strength exercise or activity (such as yoga, Pilates, resistance bands, weights, squats, or sit-ups)

Question 1: In the past 7 days, on how many days did you do strength activities for at least 10 minutes? This could include using aids that help you move, such as a wheelchair or similar equipment. ○ Days ________________________________________________

Question 2: On average, how much time did you spend doing strength activities on each day that you did the activity? ○ Minutes per day (e.g., 1.5 hours = 90 minutes) ________________________________________________

Question 3: Was the effort you put into doing strength activities usually enough to raise your breathing rate? ○ Yes ○ No

End of Block: Strength

Start of Block: Housework

Question 1: In the past 7 days, have you done any housework, gardening, or other non-exercise physical activity lasting more than 10 minutes? ○ Yes ○ No

Display This Question: If In the past 7 days, have you done any housework, gardening, or other non-exercise physical activity lasting more than 10 minutes? = Yes

Question 2: In the past 7 days, on how many days did you do housework, gardening, or other non-exercise physical activity? ○ Days ________________________________________________

Question 3: On average, how much time did you spend doing housework, gardening, or other non-exercise physical activity on each day that you did the activity? ○ Minutes per day (e.g., 1.5 hours = 90 minutes) ________________________________________________

Question 4: Was the effort you put into housework, gardening, or other non-exercise physical activity usually enough to raise your breathing rate? ○ Yes ○ No

End of Block: Housework

Start of Block: Sedentary

The next questions are about the time you spent sitting during the last 7 days. Please include time spent at work, at home, while studying, and during leisure time. This may include time sitting at a desk, in a car or on public transport, visiting friends, reading, or sitting/lying down to watch television.

Question 1: During the past 7 days, on average, how much time did you spend sitting on a weekday? Please round up to the nearest 15 minutes:

15 minutes = 0.25 hours

30 minutes = 0.50 hours

45 minutes = 0.75 hours

For example, 6 hours and 30 minutes of sitting time would be recorded as 6.5 hours. ○ Hours per day ________________________________________________

Question 2: On how many weekdays do you usually sit for this long? ○ 1 day ○ 2 days ○ 3 days ○ 4 days ○ 5 days

Question 3: During the past 7 days, on average, how much time did you spend sitting on a weekend day? Please round up to the nearest 15 minutes (same rounding method as above). For example, 6 hours and 30 minutes of sitting time would be recorded as 6.5 hours. ○ Hours per day ________________________________________________

Question 4: On how many weekend days do you usually sit this long? ○ 1 day ○ 2 days

End of Block: Sedentary

Start of Block: Thoughts on Physical Activity

How much do you agree or disagree with the following statements about doing physical activity and exercise?

Statement Strongly Agree Agree a Little Neither Agree nor Disagree Disagree a Little Strongly Disagree

I don’t know how to exercise.○○○○○

I’m worried I’ll get sweaty.○○○○○

I’m worried I’ll hurt or injure myself.○○○○○

Exercising makes me feel confident.○○○○○

Exercising makes me feel happy.○○○○○

I feel better about my body when I exercise.○○○○○

I feel healthier when I exercise.○○○○○

I find exercise boring.○○○○○

I feel physical pain when I exercise.○○○○○

I’m too busy to exercise.○○○○○

I’m worried about what people will think if they see me exercising.○○○○○

I’m worried about trying something new.○○○○○

I’m not sure how it will benefit me.○○○○○

End of Block: Thoughts on Physical Activity

Start of Block: Alcohol 1

How many alcoholic drinks do you generally consume each week?

Please enter the number of each drink consumed on each day of the week in the corresponding boxes. Read each option carefully - some drinks are listed multiple times with different measurements so you don’t have to calculate them yourself. Simply enter the number of drinks in whichever options apply to you. If you don’t generally drink alcohol, please leave this section blank.

Drink Type Monday Tuesday Wednesday Thursday Friday Saturday Sunday

Beer, lager, or cider (pints or large 500 ml bottles per week)

Beer, lager, or cider (half pints or standard 330 ml bottles per week)

Beer, lager, or cider (standard 440 ml cans per week)

Wine (small 125 ml glasses per week)

Wine (medium 175 ml glasses per week)

Wine (large 250 ml glasses per week)

Spirits / liqueurs (30% ABV or more, singles 25 ml per week)

Lower-alcohol spirits / liqueurs (< 30% ABV, e.g., Baileys, Tia Maria, singles 25 ml per week)

Alcopops (standard 275 ml bottles per week)

End of Block: Alcohol 1

Start of Block: Alcohol 2

Question: How often would you purposefully swap an alcoholic drink for a non-alcoholic or lower-alcoholic alternative? (For example, swapping to a non-alcoholic beer or soft drink, or swapping wine for a wine spritzer, or lager for a lager shandy.)

○ Never ○ Very occasionally ○ Some of the time ○ Most of the time ○ Always ○ Not applicable

End of Block: Alcohol 2

Start of Block: Alcohol 3

How much do you agree or disagree with each of the following statements?

Statement Strongly Agree Agree a Little Neither Agree nor Disagree Disagree a Little Strongly Disagree Not Applicable

I avoid going out for social drinks when I am trying to manage my weight.○○○○○○

I find it easier not to drink alcohol at all when I am trying to manage my weight.○○○○○○

When I drink alcohol, I find it more difficult to manage my eating habits.○○○○○○

I used to find it difficult to manage drinking alcohol socially, but since joining Slimming World I feel more in control.○○○○○○

I generally manage alcohol within my daily Syns allowance.○○○○○○

End of Block: Alcohol 3

Start of Block: Alcohol 4

Below is a selection of foods and alcoholic drinks. Please put these in order depending on how many calories you think each option contains - with the lowest-calorie option at the top (number 1) and the highest-calorie content at the bottom (number 7).

______ 2 pints of cider ______ 2-finger KitKat ______ McDonald’s cheeseburger ______ 1 large glass of red wine ______ 1 packet of crisps ______ Double shot of vodka with diet mixer ______ Standard-size Mars bar

End of Block: Alcohol 4

Start of Block: Wider Determinants 1

Question: Is there anyone who lives at home with you? (Please tick all that apply.)

▢ ⊗ No, just myself ▢ A partner ▢ A child/children under the age of 2 years ▢ A child/children aged 2-15 years ▢ A young person(s) aged 16-21 years ▢ Parent(s) ▢ Other family member(s), please specify (e.g., any children over 21, grandparent, nephew/niece, etc.)

▢ Other non-family member(s), please specify (e.g., friend, housemate)

Display This Question: If Is there anyone who lives at home with you? ≠ No, just myself

Who in your household has the most influence over what food is bought and eaten? ○ Me ○ My partner ○ Parent(s) ○ Other family member(s), please specify: ________________________________________________ ○ Other non-family member(s), please specify: ________________________________________________

Display This Question: If Is there anyone who lives at home with you? ≠ No, just myself

Do members of your household usually eat the same meals as you when you’re in the house together? (Please tick all that apply.)

▢ ⊗ No, we usually eat different meals ▢ My partner does ▢ My child/children do ▢ My parent(s) do ▢ Other family member(s) do, please specify: ________________________________________________ ▢ Other non-family member(s) do, please specify: ________________________________________________

End of Block: Wider Determinants 1

Start of Block: Wider Determinants 2

Display This Question: If Is there anyone who lives at home with you? (Please tick all that apply.) ≠ No, just myself

Question: Is anybody else living in your household currently a Slimming World member? (Please tick all that apply.)

▢ My partner ▢ A child/children aged 11-15 years ▢ A young person(s) aged 16-21 years ▢ Parent(s) ▢ Other family member(s), please specify (e.g., any children over 21, grandparent, nephew/niece, etc.)

▢ Other non-family member(s), please specify (e.g., friend, housemate)

▢ ⊗ No

End of Block: Wider Determinants 2

Start of Block: Wider Determinants 3

Display This Question: If Is there anyone who lives at home with you? (Please tick all that apply.) ≠ No, just myself

Question 1: Because of your Slimming World membership, do any members of your household now eat healthier meals? (Please tick all that apply.)

▢ My partner ▢ My child/children ▢ Parent(s) ▢ Other family member(s), please specify (e.g., any children over 21, grandparent, nephew/niece, etc.)

▢ Other non-family member(s), please specify (e.g., friend, housemate)

▢ ⊗ No

Question 2: Since joining Slimming World, do you feel you have influenced other people to make their own healthier food choices because of your knowledge of Food Optimising? (Please tick all that apply.)

▢ My partner ▢ My child/children ▢ Parent(s) ▢ Other family member(s), please specify: ________________________________________________ ▢ Friend(s) ▢ Work colleague(s) ▢ Other, please specify: ________________________________________________ ▢ ⊗ No

Question 3: Have you influenced anyone else to join Slimming World? (Please tick all that apply.)

▢ My partner ▢ My child/children ▢ Parent(s) ▢ Other family member(s), please specify: ________________________________________________ ▢ Friend(s) ▢ Work colleague(s) ▢ Other non-family member(s), please specify: ________________________________________________ ▢ ⊗ No

Display This Question: If Is there anyone who lives at home with you? (Please tick all that apply.) ≠ No, just myself

Question 4: Because of your Slimming World membership, has anyone in your household now become more active? (Please tick all that apply.)

▢ My partner ▢ My child/children ▢ Parent(s) ▢ Other family member(s), please specify: ________________________________________________ ▢ Other non-family member(s), please specify: ________________________________________________ ▢ ⊗ No

End of Block: Wider Determinants 3

Start of Block: Debrief

Display This Question: If Please tell us if you’re an online or group member = Group member

You have completed the survey. Thank you for taking the time to answer these questions. We really appreciate your participation and would like to remind you that all the answers you have provided will be anonymised and remain strictly confidential.

As part of our research, we would like to contact you to take part in follow-up surveys as you progress on your Slimming World journey. We will do this using the membership number and email address you provided at the start of the survey.

Please verify that your details below are correct:

Email address: ${Q71/ChoiceTextEntryValue} Membership number: ${Q2/ChoiceTextEntryValue}

○ Yes, my details are correct ○ No, my details are incorrect

Display This Question: If You have completed the survey… = No, my details are incorrect

Thank you for letting us know. Please enter your correct details below:

○ Email address ________________________________________________ ○ Membership number ________________________________________________

Display This Question: If Please tell us if you’re an online or group member = Online member

You have completed the survey. Thank you for taking the time to answer these questions. We really appreciate your participation and would like to remind you that all the answers you have provided will be anonymised and remain strictly confidential.

As part of our research, we would like to contact you to take part in follow-up surveys as you progress on your Slimming World journey. We will do this using the email address you provided at the start of the survey.

Please verify that your details below are correct:

Email address: ${Q71/ChoiceTextEntryValue}

○ Yes, my details are correct ○ No, my details are incorrect

Display This Question: If You have completed the survey… = No, my details are incorrect

Thank you for letting us know. Please enter your correct email address below:

○ Email address ________________________________________________

Prize Draw Section As a thank you for taking part in our research, we are offering all members the opportunity to take part in a prize draw for each survey. This final prize draw gives you the chance to win:

One £250 voucher

One £100 voucher

Or one of three £50 vouchers

Please indicate below if you would like to be entered into the prize draw:

○ Yes, I would like to enter the prize draw ○ No, I do not want to take part in the prize draw

End of Block: Debrief
